# *Abiotrophia defectiva* DnaK Promotes Fibronectin-Mediated Adherence to HUVECs and Induces a Proinflammatory Response

**DOI:** 10.3390/ijms22168528

**Published:** 2021-08-08

**Authors:** Minoru Sasaki, Yu Shimoyama, Yoshitoyo Kodama, Taichi Ishikawa

**Affiliations:** Division of molecular microbiology, Department of Microbiology, Iwate Medical University, 1-1-1 Idaidori, Yahaba-cho, Shiwa-gun, Iwate 028-3694, Japan; yushimo@iwate-med.ac.jp (Y.S.); microbio@catv-mic.ne.jp (Y.K.); tishikaw@iwate-med.ac.jp (T.I.)

**Keywords:** *Abiotrophia defectiva*, DnaK, fibronectin-binding protein, HUVECs, proinflammatory response

## Abstract

*Abiotrophia defectiva* is a nutritionally variant streptococci that is found in the oral cavity, and it is an etiologic agent of infective endocarditis. We have previously reported the binding activity of *A. defectiva* to fibronectin and to human umbilical vein endothelial cells (HUVECs). However, the contribution of some adhesion factors on the binding properties has not been well delineated. In this study, we identified DnaK, a chaperon protein, as being one of the binding molecules of *A. defectiva* to fibronectin. Recombinant DnaK (rDnaK) bound immobilized fibronectin in a concentration-dependent manner, and anti-DnaK antiserum reduced the binding activity of *A. defectiva* with both fibronectin and HUVECs. Furthermore, DnaK were observed on the cell surfaces via immune-electroscopic analysis with anti-DnaK antiserum. Expression of IL-8, CCL2, ICAM-1, and VCAM-1 was upregulated with the *A. defectiva* rDnaK treatment in HUVECs. Furthermore, TNF-α secretion of THP-1 macrophages was also upregulated with the rDnaK. We observed these upregulations in rDnaK treated with polymyxin B, but not in the heat-treated rDnaK. The findings show that *A. defectiva* DnaK functions not only as an adhesin to HUVECs via the binding to fibronectin but also as a proinflammatory agent in the pathogenicity to cause infective endocarditis.

## 1. Introduction

*Abiotrophia defectiva*, which is part of the nutritionally variant streptococci along with *Granulicatella adiacens*, was first described by Frenkel and Hirsch [1] on the basis of its characteristic growth requirements. It is found in the oral cavity along with other streptococci. Takeshita et al. [2] have reported that among the oral commensal bacteria, *Abiotrophia* is an early colonizer of tooth surfaces. Adhesion factors are required for bacteria to settle in a niche. There have been various studies of oral streptococci, including sialic acid-binding protein in *Streptococcus sangunis* [3] and PAc in *S. mutans* [4]. Recently, Sasaki et al. [5] suggested that *A. defectiva* colonize in oral cavity using the glyceraldehyde-3-phosphate dehydrogenase (GAPDH) to salivary pellicle proline-rich proteins. Elucidating the adhesion mechanisms of a bacterium can clarify not only the characteristics of the bacterial flora at various body sites but also the pathogenicity of the bacterium in infectious diseases. Bacterial adhesion to host tissues is thought to be a critical early step in infection. Sasaki et al. [6] and Senn et al. [7] reported the binding activity of *A. defectiva* to fibronectin and to human umbilical vein endothelial cells (HUVECs), but the adhesion mechanisms involved are not clear. Fibronectin found in the extracellular matrix of connective tissues and in the body fluid, including saliva and blood, is considered to be an important ligand involved in the binding of Gram-positive cocci to host tissues [8,9]. Consequently, fibronectin-mediated bacterial adherence to host tissues is a key process in bacterial infection. However, the contribution of some adhesion factors on the binding properties has not been well delineated. In this study, we have identified DnaK as the potential molecule binding *A. defectiva* to fibronectin. DnaK is one of the chaperon proteins that acts as polyfunctional moonlighting proteins [10,11,12]. We have also assessed the binding activity of *A. defectiva* to HUVECs using anti-DnaK antiserum to study the association of DnaK with adherence to endothelia cells via fibronectin. Through immune-electroscopic analysis, we have further confirmed DnaK of *A. defectiva* to be located on the bacterial surface. These findings indicate that DnaK is a surface adhesin that facilitates binding to host cells. On the other hand, some heat-shock proteins have been revealed to stimulate the immune system to produce cytokines or chemokines [13,14,15]. It has been reported that the expression of cell adhesion molecules such as ICAM-1 and VCAM-1 in HUVEC is rapidly and strongly induced by TNF-α [16,17]. In addition, Guimaraes et al. showed that prognostic modeling identified interleukin (IL)-8 and CCL2 as the strongest individual predictors of mortality in bacteremia [18]. These cytokines and chemokines induce leukocyte accumulation and adhesion molecule expression, and they are considered to be the most important for tissue infiltration of leukocytes. Therefore, the critical ability of proinflammatory response of DnaK was investigated in relation to the expressions of IL-8, CCL2, ICAM-1, and VCAM-1 in HUVECs and TNF-α secretion in THP-1 macrophages. The findings show that *A. defectiva* DnaK could function not only as an adhesin to HUVECs via the binding to fibronectin but also as a proinflammatory agent that induces the expression of chemokine, cytokine, and adhesion molecules in the pathogenicity. The proinflammatory response of DnaK molecules in blood vessels may be severely pathogenic to cause infective endocarditis.

## 2. Results

### 2.1. Identification of Fibronectin-Binding Molecules in A. defectiva Cells with Two-Dimensional Electrophoresis and Far-Western Blotting

**Figure 1:**
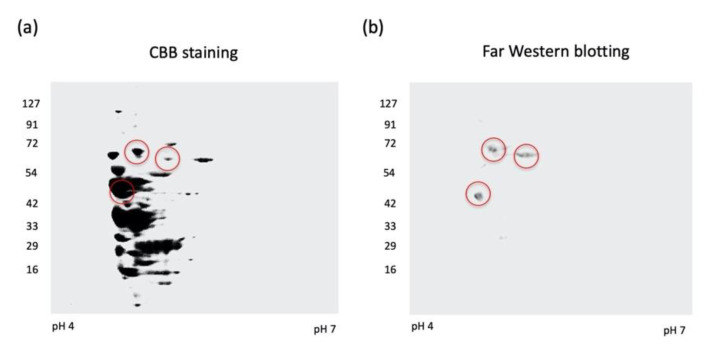
Two-dimensional electrophoresis and Far-Western blotting. Bacterial cells of *A. defectiva* ATCC (7.5 × 10^6^ CFU) were lysed and isoelectric focusing, followed by SDS-PAGE, was applied. Gel stained with Coomassie Brilliant Blue (CBB) (**a**) and the membrane blotted protein was incubated at 4 °C overnight with 50 µg/mL of fibronectin and then probed with rabbit anti-fibronectin antibodies diluted at 1:500 in PBS (pH 7.0). The membrane was then washed with PBST and incubated with a 1:2500 dilution of goat anti-rabbit IgG conjugated with HRP (**b**).

To study the bacterial cell components that can adhere to fibronectin, we analyzed *A. defectiva* cells using two-dimensional electrophoresis, followed by Far-Western blotting. Gel stained with CBB to visualize proteins revealed many protein spots that migrated with each preparation of bacterial cells (Figure 1a). Some proteins reacted with fibronectin, followed by anti-fibronectin antibody on transferred PVDF membrane. We observed three spots corresponding to molecular masses of 66 kDa, 63 kDa, and 47 kDa (Figure 1b).

We clearly identified the 66 kDa protein to be a molecular chaperone DnaK of *A. defectiva* using MASCOT with MS/MS peptide ions (Supplementary Figure S1). It contains 610 amino acids, and we calculated the molecular weight and the theoretical isoelectric point as 65,730 and 4.70, respectively, using the ProtParam tool (https://web.expasy.org/protparam/, accessed on 30 November 2020). Conversely, we did not identify the 63 kDa and 47 kDa protein spots as *A. defectiva* peptide sequences. Therefore, we analyzed the 66 kDa protein as a potential fibronectin-binding protein of *A. defectiva*.

### 2.2. Binding of rDnaK to Immobilized Fibronectin and the Effect of Anti-Dnak Antiserum on the Binding of A. defectiva to Immobilized Fibronectin or HUVECs

**Figure 2:**
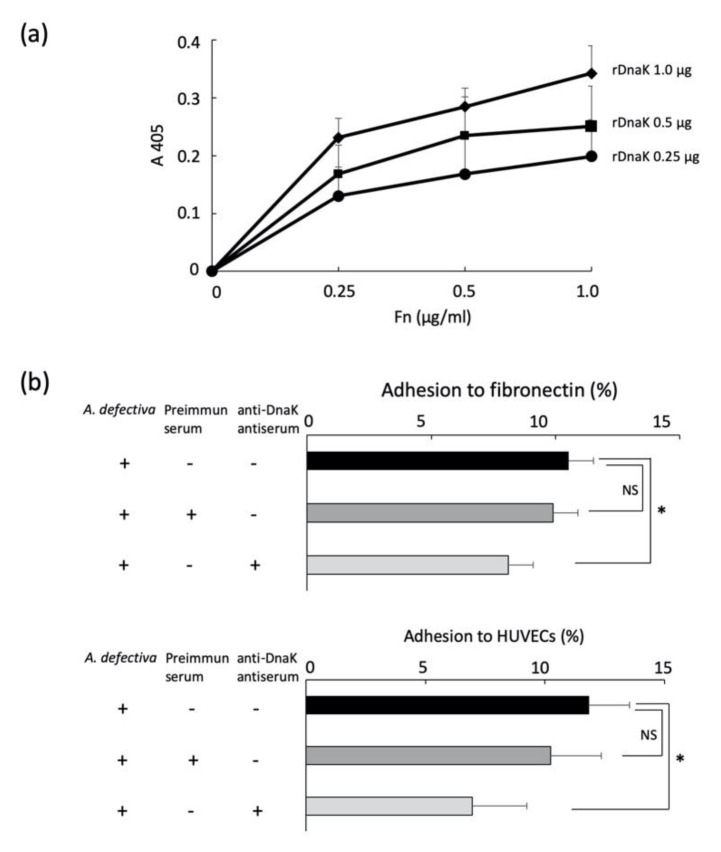
Binding of rDnaK to immobilized fibronectin, and the binding of *A. defectiva* to immobilized fibronectin or HUVECs. The indicated amounts (0.25, 0.5, and 1.0 µg/mL) of rDnaK/100 µL were added, and the fibronectin immobilized plate was incubated for 2 h at RT. The wells were washed three times with PBS, incubated with a rabbit anti-His affinity-purified antibody (1:1000) for 1 h at RT, and then incubated with an alkaline phosphatase-conjugated monoclonal anti-rabbit IgG for 1 h at RT. Alkaline phosphatase activity of each well was detected using 5 mM disodium *p*-nitrophenyl phosphate in 50 mM glycine and 0.5 mM MgCl_2_, and the absorbance at 405 nm was measured (**a**). One hundred microliters of radioactively labeled *A. defectiva* (10,000 cpm; 1 × 10^6^ CFU) were added to the wells of a 96-well microtiter plate immobilized with 2.0 µg of fibronectin. After 90 min incubation at 37 °C, the adherent bacteria were collected in a vial containing liquid scintillator and counted using a scintillation counter. The bacteria (10,000 cpm; 1 × 10^6^ CFU) were added to a 12-well plate with a semiconfluent culture of HUVECs (10^5^ cells/well) at a multiplicity of infection of 10. After 90 min incubation at 37 °C, the adherent bacteria were removed, collected, and then counted using a scintillation counter, as described previously (**b**). In some experiments, 100 µL of radioactively labeled *A. defective* that was pretreated with anti-DnaK antiserum diluted in the 1:200 or preimmune antiserum (dilution 1:200) for 1 h at RT was used. Data are expressed as the mean ± SD from three independent experiments, each performed in duplicates. Six samples were analyzed in each group. Statistically significant differences; NS, not significant, * *p* < 0.05.

The rDnaK was clearly bound to immobilized fibronectin in a concentration-dependent manner (0.25–1.0 µg/mL) (Figure 2a). We then studied the adhesion of *A. defectiva* to immobilized fibronectin or HUVECs and the effects of anti-DnaK antiserum on these binding activities. Although we recognized the adhesion of *A. defectiva* to immobilized fibronectin and HUVECs, the adhesion decreased significantly when the bacterial cells were first treated with anti- rDnaK antiserum (dilution; 1:200) (Figure 2b).

### 2.3. Localization of A. defectiva DnaK

**Figure 3:**
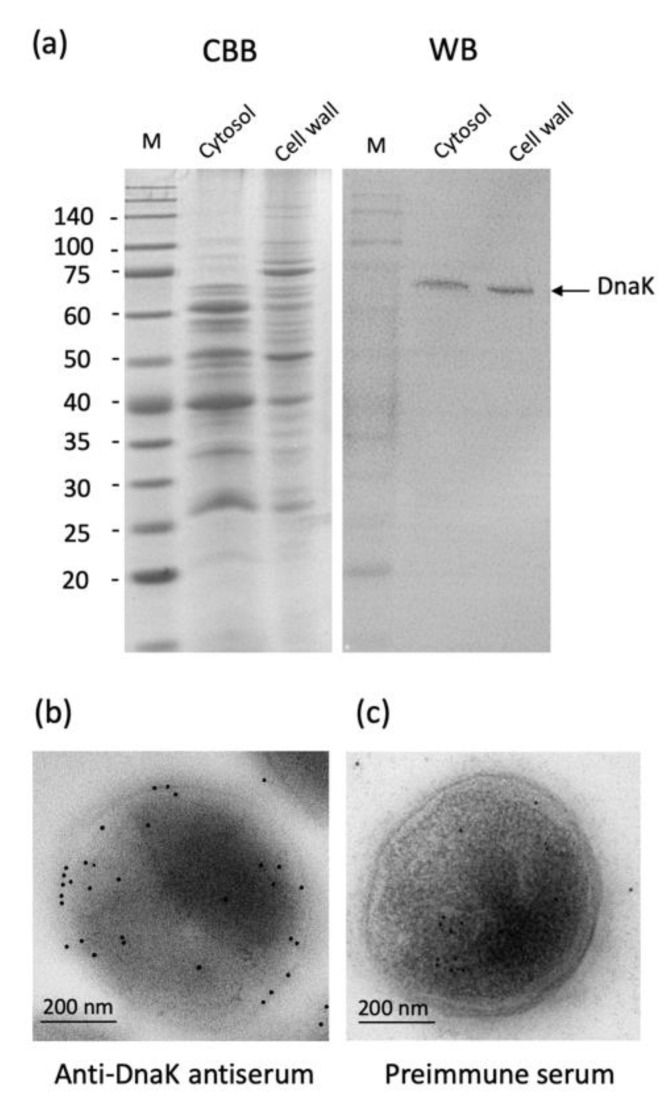
Localization of *A. defectiva* DnaK. The proteins cell lysate and cell envelope compartments were subjected to SDS-PAGE, and proteins were either stained with Coomassie Brilliant Blue (CBB) or transferred to an Immobilon-P membrane for Western blotting using the anti-DnaK antiserum (**a**). Immunoelectron microscopy was performed to confirm the localization of *A. defectiva* DnaK. The sections prepared were incubated with either rabbit anti-DnaK antiserum (**b**; dilution, 1:100) or preimmune serum (**c**; dilution, 1:100) for 48 h at 4 °C. After washing with 0.1 M TBS, the sections were incubated with 1:100 dilution of goat polyclonal anti-rabbit IgG-gold (10 nm; Abcam, Cambridge, UK) for 2 h at RT.

We studied the localization of DnaK of *A. defectiva* by Western blotting assay and immune-electroscopic analysis. The cell wall fraction and the cytoplasmic fraction prepared from *A. defectiva* containing a 66-kDa band reacted with anti-DnaK antiserum (Figure 3a). Moreover, DnaK were located on the cell surface using anti-DnaK antiserum by immune-electroscopic analysis (Figure 3b). Conversely, when we used preimmune serum, we did not detect the reacted gold particles on the cell surfaces (Figure 3c). These findings suggest that DnaK is localized on the cell surfaces of *A. defectiva*. In addition, we detected DnaK was detected in the culture medium of *A. defectiva* cells (Supplementary Figure S2), suggesting that DnaK could be present on cell surfaces and also released to bacterial culture media.

### 2.4. Cytokines and Adhesion Molecules Induction in HUVECs or THP-1 Cells Stimulated with A. defectiva rDnaK

**Figure 4:**
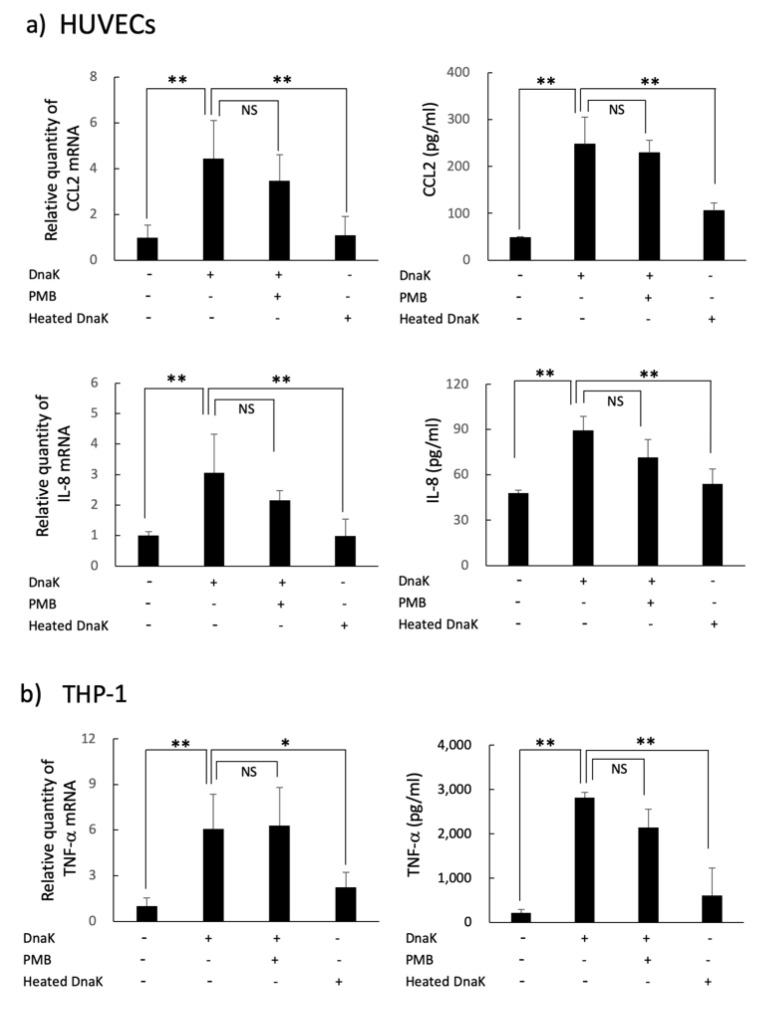
Induction of cytokines in HUVECs or THP-1 cells stimulated with *A. defectiva* rDnaK. *A. defectiva* rDnaK was added to the wells of a 24-well micro-plate with a semiconfluent culture of HUVECs (**a**) or THP-1 cells (**b**) at a dose of 2 µg/mL. After incubation for 1 or 4 h at 37 °C, total RNAs were extracted and the complementary DNA was synthesized from total RNAs. Quantitative real-time RT-PCR analysis was conducted using the primers for TNF-α, IL-8, CCL2, and GAPDH. The contents of TNF-α, IL-8, and CCL2 in culture supernatants were measured using ELISA kits. In some experiments, DnaK either treated with 10 µg/mL of PMB for 1 h or heated for 15 min at 100 °C was used. Data are expressed as the mean ± SD from three independent experiments, each performed in duplicates. Six samples were analyzed in each group. Statistically significant differences; NS, not significant, * *p* < 0.05, ** *p* < 0.01.

**Figure 5:**
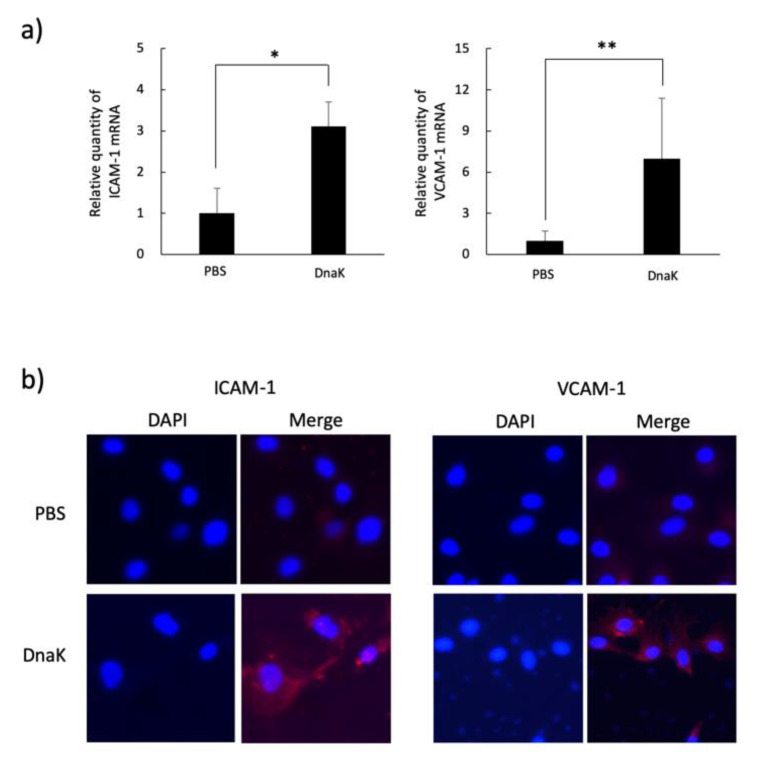
Induction of adhesion molecules in HUVECs stimulated with *A. defectiva* rDnaK. The expression of adhesion molecules on HUVECs stimulated with *A. defectiva* rDnaK was performed by the quantitative real-time RT-PCR method using the primer ICAM-1 VCAM-1 (**a**). The expression of the adhesion molecules was analyzed with an immune-staining assay using monoclonal anti-ICAM-1 antibodies (dilution; 1:200) or anti-VCAM-1 antibodies (dilution; 1:200), followed by treatment with an Alexa Fluor 594-conjugated goat anti-rabbit IgG, and the nuclei were stained with DAPI (**b**). Data are expressed as the mean ± SD from three independent experiments, each performed in duplicates. Six samples were analyzed in each group. Statistically significant differences; NS, not significant, * *p* < 0.05, ** *p* < 0.01.

Upregulation of mRNA and the protein production of CCL2, IL-8 and TNF-α were revealed by treatment with *A. defectiva* rDnaK in HUVECs and THP-1 cells, respectively (Figure 4a,b). Although the cytokine upregulation of rDnaK treated with polymyxin B (10 µg/mL) was similar to that of untreated rDnaK, it declined with heating (DnaK heated at 100 °C for 15 min). In addition, through RT-qPCR analysis and by immunostaining using anti-VCAM-1 and anti-ICAM-1 antibodies, we determined that the mRNA and protein expressions of adhesion molecules ICAM-1 and VCAM-1 of HUVECs were also upregulated with the rDnaK at a dose of 2 µg/mL (Figure 5a,b).

## 3. Discussion

Bacterial adherence to targets of epithelial or endothelial cells may be a key step in causing infection. In particular, the cell-surface ligand fibronectin is a major target of bacteria on host cells [8,9]. Many fibronectin-binding proteins of bacteria have been reported [19]. The fibronectin-binding proteins of *S. pyogenes* [20], *S. aureus* [21], *S. anginosus* [22], *S. intermedius* [23] carry binding molecules to host tissues and have been suggested as being associated with pathogenicity. In agreement with the findings of Tart and van de Rijn [24], Senn et al. [25], and Sasaki et al. [6], our findings here show that *A. defectiva* is more likely to bind to immobilized fibronectin and to HUVECs and thus might bind more readily to cells via fibronectin. However, the molecules that bind to fibronectin of *A. defectiva* have not been clarified previously. In this study, we have identified by Far-Western blotting, followed by LC–MS/MS, a putative fibronectin-binding protein, DnaK, a chaperon protein that is located on the *A. defectiva* cell surfaces.

DnaK is a bacterial member of the highly conserved, ubiquitous family of 70-kDa heat-shock-induced chaperone proteins (Hsp70 proteins), and its expression is induced by heat and other general stress responses [26,27]. Several studies suggest that the bacterial Hsp70 protein DnaK is a cell-surface protein in a growing number of bacteria and that it functions as a ligand for host proteins [10,28,29] or DNA [30]. In this study, we found that *A. defectiva* DnaK clearly binds to fibronectin and also binds to HUVEC, and this binding was suppressed by anti-DnaK antiserum. Further, it is located on the cell surfaces by the analysis of immunoelectron microscopy using anti-DnaK antiserum suggesting that this bacterium binds to HUVECs with DnaK, as the fibronectin-binding protein. Therefore, the binding molecule of *A. defectiva* could function as a critical adhesin to host proteins.

Some studies have reported the biological or immunological activities of Hsp and DnaK and have suggested that these activities could represent the pathogenicity of bacteria. *Mycobacterium tuberculosis* Cpn60 proteins show cytokine-inducing ability [31], and this is suggested as being involved in generating granulomas. Moreover, according to Kol et al. [32], *Chlamydia pneumoniae* Cpn60 in atherosclerotic plaques stimulated monocytes and induced proinflammatory cytokine and metalloproteinase synthesis. Consequently, we have also studied the association of the biological activities of *A. defectiva* rDnaK to HUVECs and THP-1 cells with pathogenicity. We recognized upregulation of mRNA and protein production of IL-8 and CCL2 in HUVECs. These findings suggest that *A. defectiva* adheres to blood vessels with cell-surface DnaK and stimulates HUVECs to produce CCL2 and IL-8 by cell-bound and soluble DnaK, resulting in the recruitment of monocytes and neutrophils. The upregulation of rDnaK expressed using LPS-eliminated *E. coli*, was descended by heat treatment; however, it was not inhibited with PMB indicating that the upregulation depends on protein component but not LPS. Furthermore, ICAM-1 plays an important role in tight adhesion and leukocyte migration from blood vessels into tissues. ICAM-1 is normally expressed on the surface of cells such as endothelial cells and immune-system cells, and its expression is upregulated by various stimuli including TNF-α, IFN-γ, and IL-1 [33]. Moreover, VCAM-1 expression is induced in endothelial cells by inflammatory cytokines, including TNF-α and IL-1β [34]. VCAM-1 on endothelial cells interacts with the integrin VLA-4 (α4β1) on leukocytes to mediate the migration of circulating leukocytes from the blood across the endothelium and into tissues [35]. In this study, we found upregulation of ICAM-1 and VCAM-1 mRNA expression and the protein expression on the surfaces of HUVECs. Moreover, for THP-1 cells, TNF-α production was induced by the stimulation with *A. defectiva* DnaK (Figure 3b). These results suggest that DnaK can directly induce adhesion molecules to HUVECs, along with cytokine upregulation, and can induce adhesion molecules indirectly through TNF-α produced from THP-1 stimulated with DnaK.

Conversely, the activation of monocytes and epithelial cells by rDnaK of either *Fransicella turallensis* or *Pseudomonas aeruginosa* has been shown to be upregulated depending on TLR4 signaling [36,37]. Our preliminary experiment revealed that pretreatment with HUVECs by TAK242, an inhibitor of TLR4 signaling, reduced the upregulation of *A. defectiva* rDnaK (Supplementary Figure S3). This indicates that *A. defectiva* DnaK—as with *P. aeruginosa* and *F. tularensis*—may also stimulate host cells through TLR4. However, the inhibition by TAK242 was not significant in THP-1 cells. The responsiveness may differ depending on the cell type. Therefore, analysis of the levels of expression of a pattern recognition receptor or a search for another receptor is required in the future. In addition, Wang et al. suggested that the ability of mycobacterial HSP70 to activate innate immunity is located in the C-terminal region of the peptide [38]. The phylogenetic tree of DnaK of *A. defectiva* and of some other bacteria that have been reported to have DnaK is shown in Supplementary Figure S4, wherein the relationship between *M. tuberculosis* and *A. defectiva* is shown to be a bit distant. Therefore, it is necessary to study the structure–activity relationship by producing truncated recombinant proteins of different lengths of DnaK to analyze the binding to immunological active sites.

Furthermore, as the intracellular/surface moonlighting proteins, in addition to DnaK, do not contain either signal sequences for secretion or known sequence motifs for binding to the cell surface, the question of how these proteins are either secreted or become attached to the cell surface has not been answered in most cases. The export of macromolecules via extracellular membrane-derived vesicles plays an important role in the physiological function of Gram-negative bacteria [39,40]. In addition, Matsunaga et al. [41] suggested that autolysin mediates the expression of GAPDH on the surface of *C. perfringens*. Therefore, there is a need to also study the secretory mechanisms of *A. defectiva* DnaK in the future.

## 4. Materials and Methods

### 4.1. Bacterial Strains and Culture Conditions

*A. defectiva* ATCC was cultured in Todd–Hewitt broth (THB; Difco Laboratories. Detroit. Mich.) supplemented with 10 μg/mL of pyridoxal hydrochloride (Wako Pure Chemicals, Tokyo, Japan) and 200 μg/mL of L-cystein hydrochloride (Kanto Chemicals, Tokyo, Japan) at 37 °C for 48 h under anaerobic conditions. In some experiments, the bacteria were cultured with 37 kBq of [*methyl*-^3^H]-thymidine (PerkinElmer Japan, Yokohama, Japan) per mL of THB containing pyridoxal and l-cysteine for radioactive labeling.

### 4.2. Cultured Cells and Culture Conditions

We purchased HUVECs from the Health Science Research Bank (Osaka, Japan). The cells were maintained in HuMedia-EG2 medium (Kurabo Ind. Ltd., Osaka, Japan) supplemented with 10% fetal calf serum, 10 ng/mL fibroblast growth factor basic, and penicillin/streptomycin solution at 37 °C in an atmosphere containing 5% CO_2_. THP-1 cells (RCB 1189; Riken Cell Bank, Tsukuba, Japan) were maintained in RPMI 1640 medium (Thermo Fisher Scientific, Roskilde, Denmark) supplemented with 10% fetal calf serum and penicillin/streptomycin solution at 37 °C in a 5% CO_2_ atmosphere. THP1 cells were differentiated using 200 nM phorbol 12-myristate 13-acetate (PMA, Sigma-Aldrich, St. Louis, MO, USA) for 3 days. The assay of proinflammatory response was performed by using serum free media.

### 4.3. Two-Dimensional Electrophoresis and Far-Western Blotting

We lysed cells of *A. defectiva* ATCC (7.5 × 10^6^ CFU) and applied isoelectric focusing, as described previously [5]. We then equilibrated the strips for 30 min in 50 mM Tris-HCl, pH 6.8, with 6 M urea, 3% *w*/*v* sodium dodecyl sulfate (SDS), 50 mM DTT, and 0.01% BPB. We performed second-dimension analysis (SDS-PAGE) using homogeneous running gels (12.5%) without a stacking gel. For immunoblot analysis, we subjected the proteins to isoelectric focusing followed by SDS-PAGE using a polyvinylidene difluoride (PVDF) membrane (Immobilon-P; Millipore, Burlington, MA, USA) at 200 mA for 1 h. We used 1% (*v*/*v*) bovine serum albumin (BSA) to block nonspecific protein binding to the membrane. After washing with phosphate-buffered saline containing 0.15% Tween 20 (PBST), the membrane was incubated overnight at 4 °C with 50 µg/mL of fibronectin (Sigma-Aldrich, St. Louis, MO, USA). After further washing with PBST, we probed the membrane with rabbit anti-fibronectin antibodies diluted at 1:500 in PBS (pH 7.0) at 4 °C for 6 h. The membrane was then washed again with PBST and incubated with 1:2500-dilution of goat anti-rabbit immunoglobulin G (IgG) conjugated with horseradish peroxidase (HRP) at 4 °C for 1 h. We used a Chemi-Lumi One Super Western Blotting Detection System (Nacalai Tesque, Kyoto, Japan) for detection.

### 4.4. Identification of a 66-kDa Protein

We identified fibronectin-binding protein spots on 2D-PAGE using LC–MS/MS, as reported previously [42]. We excised protein spots from Coomassie Brilliant Blue (CBB) G-250-stained gels. Following alkylation, these spots were digested with trypsin (Promega, Madison, WI, USA) at 37 °C overnight. We extracted peptides with 50% (*v*/*v*) acetonitrile and 2.5% (*v*/*v*) formic acid. We performed ion-trap tandem mass spectrometry on an HCT ultra (Bruker Daltonics, Bremen, Germany) following the manufacturer’s protocol. We carried out peptide mass fingerprinting using MASCOT (Matrix Science, Boston, MA, USA) with MS/MS peptide ions.

### 4.5. Construction of Expression Vector for A. defectiva Dnak Protein and Expression and Purification of rDnak

We generated a DNA fragment containing the *A. defectiva* DnaK gene by polymerase chain reaction (PCR), using *A. defectiva* chromosomal DNA as the template and with the following primers: Dnak-F, 5′-GTCAGGATCCGCAAAAATTATTGGTATTGACTTAGGG-3′ at the second alanine from the beginning, and DnaK-R, 5′-GTCAAGATCTGTCGATTTCTTCGAACTCAGCG-3′ ending 3 bp up stream (aspartic acid) from the stop codon. We digested the PCR fragment using BamHI and *Bg*12, and then cloned 10 ng of PCR product into pQE60 using a DNA ligation kit (ver.2, TaKaRa bio, Shiga, Japan). We transfected the construct to an lipopolysaccharide (LPS)-eliminated *Escherichia coli*, ClearColi™ BL21 (DE3), cells (COSMO Bio, Tokyo, Japan) carrying an expression plasmid, and were cultured overnight in Luria–Bertani broth supplemented with 50 µg/mL ampicillin at 30 °C. Recombinant protein expression was induced by adding 0.2 mM isopropyl β-d-1-thiogalactopyranoside at 30 °C for 3 h and purified from cells via TALON affinity chromatography [42].

### 4.6. Binding of rDnaK to Immobilized Fibronectin

We conducted the binding of rDnaK to immobilized fibronectin following our previous method [23]. In brief, we added fibronectin solutions (0.25 µg, 0.5 µg, and 1.0 µg) to the wells of a 96-microwell plate (flat bottom; Thermo Fisher Scientific) and immobilized them by incubating them at 4 °C overnight. We then blocked the wells with 200 µL of 1% (*w*/*v*) BSA (Sigma-Aldrich, St. Louis, MO, USA) at room temperature (RT) for 1 h. We added the indicated amounts of rDnaK (100 µL) and incubated the plate at RT for 2 h. We washed the wells three times with PBS and performed further incubation with a rabbit anti-His affinity-purified antibody (1:1000) (QED Bioscience, San Diego, CA, USA) in PBS, pH 7.4, for 1 h at RT followed by incubation with an alkaline phosphatase-conjugated monoclonal anti-rabbit IgG (Promega KK, Osaka, Japan; 1:3000) at RT for 1 h. We detected the alkaline phosphatase activity of each well by using 5 mM disodium *p*-nitrophenyl phosphate in 50 mM glycine and 0.5 mM MgCl_2_, and we measured the absorbance at 405 nm.

### 4.7. Binding of A. defectiva to Immobilized Fibronectin or HUVECs

We added 100 µL of radioactively [*methyl*-^3^ H-thymidine (37 kBq ml^−1^)] labeled *A. defectiva* (10,000 cpm; 1 × 10^6^ CFU) to the wells of a 96-well microtiter plate (Thermo Fisher Scientific) immobilized with 2.0 µg of fibronectin (Sigma-Aldrich, St. Louis, MO, USA). After 90 min of incubation at 37 °C, the microtiter plates were washed three times with PBS to remove unbound bacterial cells. We treated the adherent bacteria with 0.25% trypsin-ethylenediamine tetraacetic acid solution (Life Technologies Japan Ltd.) for 10 min at 37 °C to remove attached bacteria, and we collected these in a vial containing liquid scintillator and counted them using a scintillation counter [22]. We also added the bacteria (10,000 cpm; 1 × 10^6^ CFU) to a 12-well plate (Thermo Fisher Scientific) containing a semiconfluent culture of HUVECs (10^5^ cells/well) at a multiplicity of infection of 10. After 90 min incubation at 37 °C, the adherent bacteria were removed and collected, as mentioned above, and counted using a scintillation counter. In some experiments, we used 100 µL of radioactively labeled *A. defectiva* that was pretreated with anti-DnaK antiserum produced by Europhine Genomics (Eurofins Genomics, Co., Ltd., Tokyo, Japan) diluted in the 1:200 or preimmune antiserum (dilution 1:200) at RT for 1 h to study how the antiserum affected the binding activity of *A. defectiva* to either immobilized fibronectin or HUVECs.

### 4.8. Localization of A. defectiva DnaK

We separated the proteins isolated from *A. defectiva* into soluble and cell envelope compartments following the procedure of Holmes [43]. After subjecting each fraction (4 µg of protein) to SDS-PAGE, we either stained the proteins with CBB or transferred them to an Immobilon-P membrane (Millipore) for Western blotting using the anti-DnaK antiserum following the procedure of Kodama et al. [23]. In some experiments, we also analyzed DnaK in bacterial culture supernatant by Western blotting. We confirmed the localization of DnaK via immunoelectron microscopy. We cultured *A. defectiva* cells for 48 h at 37 °C under the conditions we have described previously [5]; we collected cells via centrifugation (5000 g × 5 min) and then washed them three times with 0.1 M tris-buffered saline (TBS). We fixed bacteria (5 × 10^7^ CFUs) in a solution containing 4% paraformaldehyde in TBS for 2 h at RT, and we then dehydrated them and embedded them in London Resin White resin. We cut ultrathin sections and blocked them with 10% goat serum for 2 h at RT. We washed the sections with 0.1 M TBS and then incubated them with rabbit anti-DnaK antiserum (dilution, 1:100) for 48 h at 4 °C. After further washing with 0.1 M TBS, we incubated the sections with a 1:100 dilution of goat polyclonal anti-rabbit IgG-gold (10 nm; Abcam, Cambridge, UK) for 2 h at RT. We washed the sections once with 0.1 M TBS and then four times with distilled water, after which we air-dried the samples and studied them under a transmission electron microscope (TEM Model H-7650, Hitachi High-Tech Co., Tokyo, Japan).

### 4.9. Induction of Cytokines and Adhesion Molecules in HUVECs or THP-1 Cells Stimulated with A. defectiva rDnaK

We added *A. defectiva* rDnaK to the wells of a 24-well micro-plate (Thermo Fisher Scientific) with semiconfluent culture of either HUVECs or THP-1 cells at a dosage of 2 µg/mL. Following 1, 2, and 4 h incubation at 37 °C, total RNAs were extracted from the cells using the RNeasy mini kit (QIAGEN, Copenhagen, Denmark) according to the manufacturer’s protocol. We synthesized the complementary DNA from total RNAs using PrimeScript RT Master Mix (TaKaRa Bio., Shiga, Japan). We performed quantitative real-time, reverse-transcription PCR (RT-PCR) analysis using TB Green Premix Ex Taq II (TaKaRa). We purchased the primers for TNF-α, IL-8, CCL2, ICAM-1, VCAM-1, and GAPDH from TaKaRa. We measured the contents of TNF-α, IL-8, and CCL2 in culture supernatants using enzyme-linked immunosorbent assay (ELISA) kits (Proteintech Group, Inc, IL, USA). In some experiments, we ensured that no trace endotoxins contributed to the observed responses by using rDnaK treated with 10 µg/mL of polymyxin B (PMB) for 1 h at RT and heated (15 min at 100 °C). In addition, we measured the expression of adhesion molecules on HUVECs stimulated with *A. defectiva* rDnaK using an immune-staining assay. In brief, we added *A. defectiva* rDnaK to the wells of an eight-part slide culture of HUVECs at a dosage of 2 µg/mL. After 12 h incubation at 37 °C, the cells were fixed with paraformaldehyde. After washing the cells with PBS and then blocking them with 1% BSA, we treated the cells with monoclonal anti-ICAM-1 antibodies (Abcam, Tokyo, Japan; dilution; 1:200) or anti-VCAM-1 antibodies (Abcam; dilution; 1:200) overnight at 4 °C. After washing the cells with PBS, we treated them with an Alexa Fluor 594-conjugated goat anti-rabbit IgG (1:1000, Thermo Fisher Scientific KK, Tokyo, Japan) and stained the nuclei with DAPI (1:1000, Dojindo Molecular Technologies, Inc., Kumamoto, Japan). We analyzed the treated cells using fluorescence microscopy (KEYENCE, Osaka, Japan).

### 4.10. Statistical Analysis

The statistical analyses were conducted using the Student’s *t*-test and analysis of variance with Bonferroni’s post-test using MacTKV3 software (Esumi. co. jp, Tokyo, Japan). All values are expressed as the mean ± standard deviation. We considered a probability (*p*) value of <0.05, <0.01 as statistically significant.

## 5. Conclusions

In conclusion, this study has investigated the mechanisms of adhesion of DnaK located on the cell surfaces of *A. defectiva* to HUVECs and THP-1 cells and the associated biological activities of *A. defectiva* DnaK. The findings have identified *A. defectiva* DnaK to be one of the fibronectin-binding proteins associated with the binding to HUVECs. Furthermore, the findings indicate that the *A. defectiva* rDnaK induce the mRNA of chemokine, cytokine, and adhesion molecule expressions by HUVECs and THP-1 cells. Additionally, rDnaK also upregulated the expressions of ICAM-1, VCAM-1 on HUVECs. The activities of *A. defectiva* DnaK can be used to explain the pathogenicity of the bacteria in infective endocarditis. This antigen may be a target in the development of a vaccine for infective endocarditis in the future.

## Data Availability

All data generated or analyzed during this study are included in this article.

## Supplementary Materials

The following are available online at https://www.mdpi.com/article/10.3390/ijms22168528/s1. Figure S1: Identification of DnaK of *A. defectiva* by LC–MS/MS. Figure S2: The detection of DnaK in culture supernatant. Figure S3: The effect of TAK242 on the activities of DnaK on HUVECs and THP-1 cells. Figure S4: Similarities among DnaK amino acid sequences from bacterial species as per the algorithms.

## References

[1] Frenkel A., Hirsch W. (1961). Spontaneous development of L forms of streptococci requiring secretions of other bacteria or sulphydryl compounds for normal growth. Nature.

[2] Takeshita T., Yasui M., Shibata Y., Furuta M., Saeki Y., Eshima N., Yamashita Y. (2015). Dental plaque development on a hydroxyapatite disk in young adults observed by using a barcoded pyrosequencing approach. Sci. Rep..

[3] Murray P.A., Levine M.J., Reddy M.S., Tabak L.A., Bergey E.J. (1986). Preparation of a sialic acid-binding protein from *Streptococcus mitis* KS32AR. Infect. Immun..

[4] Jenkinson H.F., Demuth D.R. (1997). Structure, function and immunogenicity of streptococcal antigen I/II polypeptides. Mol. Microbiol..

[5] Sasaki M., Kodama Y., Shimoyama Y., Ishikawa T., Tajika S., Kimura S. (2020). *Abiotrophia defectiva* adhere to saliva-coated hydroxyapatite beads via interactions between salivary proline-rich-proteins and bacterial glycerlaldehyde-3-phosphate dehydrogenase. Microbiol. Immunol..

[6] Sasaki M., Shimoyama Y., Ishikawa T., Kodama Y., Tajika S., Kimura S. (2020). Contribution of different adherent properties of *Granulicatella adiacens* and *Abiotrophia defectiva* to their associations with oral colonization and the risk of infective endocarditis. J. Oral Sci..

[7] Senn L., Entenza J.M., Prod’hom G. (2006). Adherence of *Abiotrophia defectiva* and *Granulicatella* species to fibronectin: Is there a link with endovascular infections?. FEMS Immunol. Med. Microbiol..

[8] Proctor R.A. (1987). The staphylococcal fibronectin receptor: Evidence for its importance in invasive infections. Rev. Infect. Dis..

[9] Hanski E., Caparon M. (1992). Protein F, a fibronectin-binding protein, is an adhesin of the group A *streptococcus pyogenes*. Proc. Natl. Acad. Sci. USA.

[10] Xolalpa W., Vallecillo A.J., Lara M., Mendoza-Hernandez G., Comini M., Spallek R., Singh M., Espitia C. (2007). Identification of novel bacterial plasminogen-binding proteins in the human pathogen *Mycobacterium tuberculosis*. Proteomics.

[11] Kalayoglu M.V., Morrison R.P.I., Morrison S.G., Yuan Y., Byrne G.I. (2000). Chlamydial virulence determinants in atherogenesis: The role of chlamydial lipopolysaccharide and heat shock protein 60 in macrophage-lipoprotein interactions. J. Infect. Dis..

[12] Kamiya S., Yamaguchi H., Osaki T., Taguchi H. (1998). A virulence factor of Helicobacter pylori: Role of heat shock protein in mucosal inflammation after H. pylori infection. J. Clin. Gastroenterol..

[13] Wang Y., Kelly C.G., Karttunen J.T., Whittall T., Lehner P.J., Duncan L., MacAry P., Younson J.S., Singh M., Oehlmann W. (2001). CD40 is a cellular receptor mediating mycobacterial heat shock protein 70 stimulation of CC-chemokines. Immunity.

[14] Zhao Y., Yokota K., Ayada K., Yamamoto Y., Okada T., Shen L., Oguma K. (2007). Helicobacter pylori heat-shock protein 60 induces interleukin-8 via a toll-like receptor (TLR) 2 and mitogen-activated protein (MAP) kinase pathway in human monocytes. J. Med. Microbiol..

[15] Tabona P., Reddi K., Khan S., Nair S.P., Crean S.J., Meghji S., Wilson M., Preuss M., Miller A.D., Poole S. (1998). Homogeneous *Escherichia coli* chaperonin 60 induces IL-1 and IL-6 gene expression in human monocytes by a mechanism independent of protein conformation. J. Immunol..

[16] Roux M.E., Lecoq D., Meyer D., Dosne A.M. (1997). Requirement of prestimulated THP-1 monocytic cells for endothelial cell activation. Involvement of TNF alpha. Blood Coagul. Fibrinolysis.

[17] Chang C.C., Chu C.F., Wang C.N., Wu H.T., Bi K.W., Pang J.H., Huang S.T. (2014). The anti-atherosclerotic effect of tanshinone IIA is associated with the inhibition of TNF-α-induced VCAM-1, ICAM-1 and CX3CL1 expression. Phytomedicine.

[18] Guimaraes A.O., Cao Y., Hong K., Mayba O., Peck M.C., Gutierrez J., Ruffin F., Carrasco-Triguero M., Dinoso J.B., Clemenzi-Allen A. (2019). A prognostic model of persistent bacteremia and mortality in complicated *Staphylococcus aureus* bloodstream infection. Clin. Infect. Dis..

[19] Schwarz-Linek U., Höök M., Potts J.R. (2007). The molecular basis of fibronectin-mediated bacterial adherence to host cells. Mol. Microbiol..

[20] Yamaguchi M., Terao Y., Kawabata S. (2013). Pleiotropic virulence factor–*Streptococcus pyogenes* fibronectin-binding proteins. Cell Microbiol..

[21] Herman-Bausier P., El-Kirat-Chatel S., Foster T.J., Geoghegan J.A., Dufrêne Y.F. (2015). *Staphylococcus aureus* fibronectin-binding protein A mediates cell-cell adhesion through low-affinity homophilic bonds. mBio.

[22] Kodama Y., Ishikawa T., Shimoyama Y., Sasaki D., Kimura S., Sasaki M. (2018). The fibronectin-binding protein homologue Fbp62 of *Streptococcus anginosus* is a potent virulence factor. Microbiol. Immunol..

[23] Kodama Y., Shimoyama Y., Ishikawa T., Kimura S., Sasaki M. (2020). Characterization and pathogenicity of fibronectin binding protein FbpI of *Streptococcus intermedius*. Arch. Microbiol..

[24] Tart R.C., van de Rijn I. (1993). Identification of the surface component of *Streptococcus defectivus* that mediates extracellular matrix adherence. Infect. Immun..

[25] Senn L., Entenza J.M., Greub G., Jaton K., Wenger A., Bille J., Calandra T., Prod’hom G. (2006). Bloodstream and endovascular infections due to *Abiotrophia defectiva* and *Granulicatella* species. BMC Infect. Dis..

[26] Jayaraman G.C., Burne R.A. (1995). DnaK expression in response to heat shock of *Streptococcus mutans*. FEMS Microbiol. Lett..

[27] Hecker M., Schumann W., Völker U. (1996). Heat-shock and general stress response in *Bacillus subtilis*. Mol. Microbiol..

[28] Schaumburg J., Diekmann O., Hagendorff P., Bergmann S., Rohde M., Hammerschmidt S., Jänsch L., Wehland J., Kärst U. (2004). The cell wall subproteome of Listeria monocytogenes. Proteomics.

[29] Floto R.A., MacAry P.A., Boname J.M., Mien T.S., Kampmann B., Hair J.R., Huey O.S., Houben E.N., Pieters J., Day C. (2006). Dendritic cell stimulation by mycobacterial Hsp70 is mediated through CCR5. Science.

[30] Basu D., Khare G., Singh S., Tyagi A., Khosla S., Mande S.C. (2009). A novel nucleoid-associated protein of *Mycobacterium tuberculosis* is a sequence homolog of GroEL. Nucleic Acids Res..

[31] Hu Y., Henderson B., Lund P.A., Tormay P., Ahmed M.T., Gurcha S.S., Besra G.S., Coates A.R. (2008). A *Mycobacterium tuberculosis* mutant lacking the groEL homologue cpn60.1 is viable but fails to induce an inflammatory response in animal models of infection. Infect. Immun..

[32] Kol A., Sukhova G.K., Lichtman A.H., Libby P. (1998). Chlamydial heat shock protein 60 localizes in human atheroma and regulates macrophage tumor necrosis factor-alpha and matrix metalloproteinase expression. Circulation.

[33] Kuppner M.C., van Meir E., Hamou M.F., de Tribolet N. (1990). Cytokine regulation of intercellular adhesion molecule-1 (ICAM-1) expression on human glioblastoma cells. Clin. Exp. Immunol..

[34] McHale J.F., Harari O.A., Marshall D., Haskard D.O. (1999). TNF-alpha and IL-1 sequentially induce endothelial ICAM-1 and VCAM-1 expression in MRL/lpr lupus-prone mice. J. Immunol..

[35] Klemke M., Weschenfelder T., Konstandin M.H., Samstag Y. (2007). High affinity interaction of integrin alpha4beta1 (VLA-4) and vascular cell adhesion molecule 1 (VCAM-1) enhances migration of human melanoma cells across activated endothelial cell layers. J. Cell Physiol..

[36] Jeon J., Lee Y., Yu H., Ha U.H. (2020). HSP70-homolog DnaK of *Pseudomonas aeruginosa* increases the production of IL-27 through expression of *EBI3* via TLR4-dependent NF-κB and TLR4-independent Akt signaling. Int. J. Mol. Sci..

[37] Ashtekar A.R., Zhang P., Katz J., Deivanayagam C.C., Rallabhandi P., Vogel S.N., Michalek S.M. (2008). TLR4-mediated activation of dendritic cells by the heat shock protein DnaK from *Francisella tularensis*. J. Leukoc. Biol..

[38] Wang Y., Kelly C.G., Singh M., Wang Y., Kelly C.G., Singh M., McGowan E.G., Carrara A.S., Bergmeier L.A., Lehner T. (2002). Stimulation of Th1-polarizing cytokines, C-C chemokines, maturation of dendritic cells, and adjuvant function by the peptide binding fragment of heat shock protein 70. J. Immunol..

[39] Rivera J., Cordero R.J., Nakouzi A.S., Frases S., Nicola A., Casadevall A. (2010). *Bacillus anthracis* produces membrane-derived vesicles containing biologically active toxins. Proc. Natl. Acad. Sci. USA.

[40] Veith P.D., Chen Y.Y., Gorasia D.G., Chen D., Glew M.D., O’Brien-Simpson N.M., Cecil J.D., Holden J.A., Reynolds E.C. (2014). *Porphyromonas gingivalis* outer membrane vesicles exclusively contain outer membrane and periplasmic proteins and carry a cargo enriched with virulence factors. J. Proteome Res..

[41] Matsunaga N., Shimizu H., Fujimoto K., Watanabe K., Yamasaki T., Hatano N., Tamai E., Katayama S., Hitsumoto Y. (2018). Expression of glyceraldehyde-3-phosphate dehydrogenase on the surface of *Clostridium perfringens* cells. Anaerobe.

[42] Shimoyama Y., Ishikawa T., Kodama Y., Kimura S., Sasaki M. (2020). Tyrosine tRNA synthetase as a novel extracellular immunomodulatory protein in *Streptococcus anginosus*. FEMS Microbiol. Lett..

[43] Holmes A.R., McNab R., Millsap K.W., Rohde M., Hammerschmidt S., Mawdsley J.L., Jenkinson H.F. (2001). The pavA gene of *Streptococcus pneumoniae* encodes a fibronectin-binding protein that is essential for virulence. Mol. Microbiol..

