# Supplementary material for: Abiotrophia defectiva DnaK Promotes Fibronectin-Mediated Adherence to HUVECs and Induces a Proinflammatory Response
Source: Int J Mol Sci. 2021 Aug 8;22(16):8528. doi: 10.3390/ijms22168528 (PMC8395199; doi:10.3390/ijms22168528)
Supplement: Supplementary file 1 [file ijms-22-08528-s001.zip › ijms-1328250-supplementary.pdf]

MAKIIGIDLGTTNSAVSVLEGGEAK**IIPNP**
EGNRTTPSVVAFKNGEIQVGEVAKRQAVTN
PSTVSSIKRHMGDGSYKVHMDGKDYTPQEI
SAMILQYLK**SYAEDYLGEK**VDK**AVITVPAY**
FNDAQRQATKDAGKIAGLEVER**IVNEPTAA**
ALAYGLDKTDKEEKVLVFDLGGGTFDVSIL
ELGDGVFDVLSTSGDNHLGGDDFDQKIMDY
LVAEFKKEHGVDLSKDKMALQRLKDAAEKA
KKDLSGVSTTQISLPFITASAEGPLHLELT
LTRAK**FEELTHDLVER**TKQPVRQALKDAGL
SQSDIDEVILVGGSTRIPAVVEAVRKETGK
EPNKSVNPDEVVAMGAAIQGGVISGDVKDI
VLLDVTPLSLGIETMGGVFTKLIDRNTTIP
TSK**SQVFSTAADNQPAVDVHVLQGER**QMAA
DNKTLGR**FQLTDIPPAPRGIPQIEVTFDID**
KNGIVNVSAKDLGTGKEQTITIK**SSSGLTD**
EEIDRMVKDAEANAEADKKRREEADLR**NEV**
DQLVFQTDKVLADLKDKVSEEEVKKAEAAR
DELKAAIEANDLDQMKAKRDALNEIVQNLT
VKLYEQAAAQAQAAQGNGQAQADTNASGDD
VVDAEFEEID

Figure S1. Identification of DnaK of *A. defectiva* by LC–MS/MS. The protein reacted with anti-DnaK antiserum was excised from Coomassie brilliant blue stained gel. Following alkylation, spots were digested with trypsin. 11 peptide sequences marked in bold were predicted with MALDI-TOF MS. The predicted MS spectra were fingerprinted with MS-Fit.

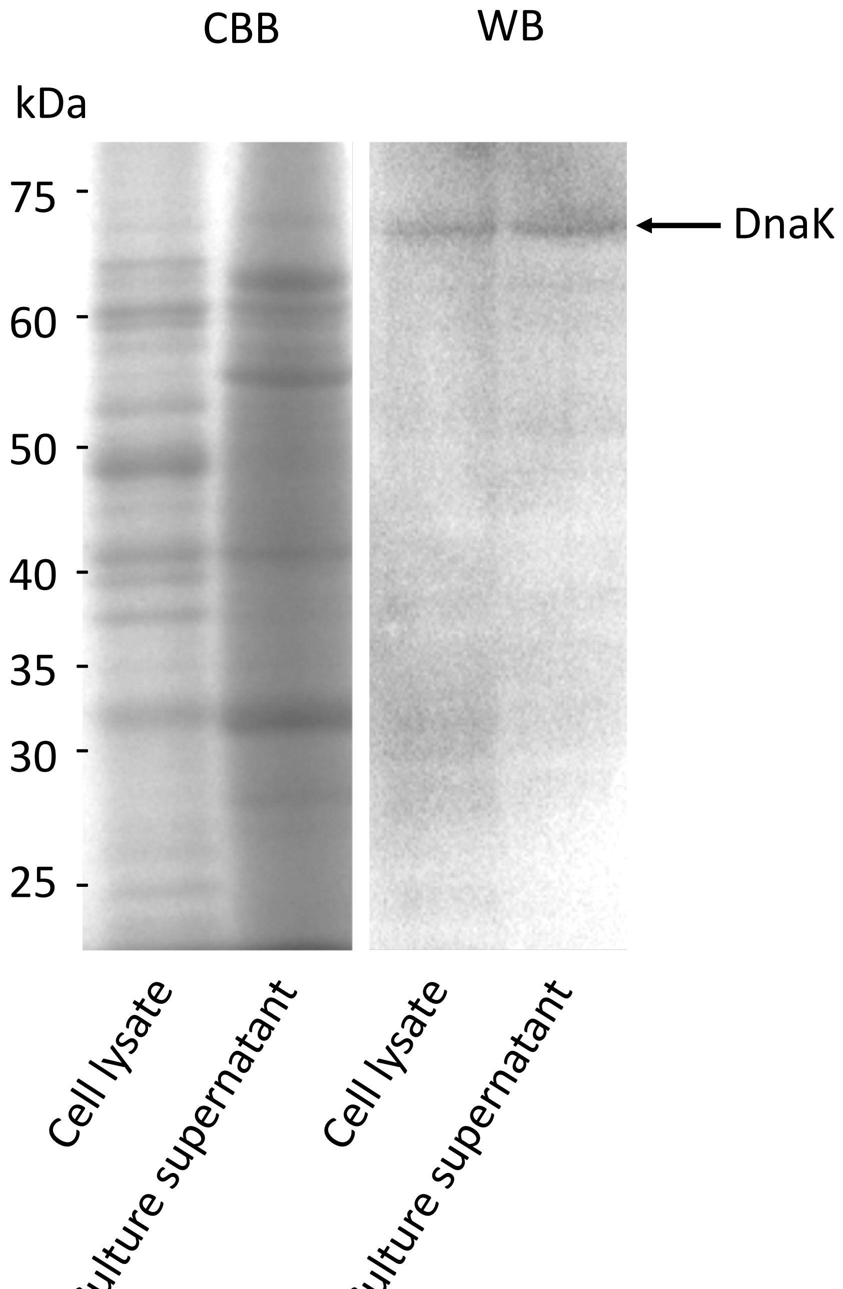

Figure S2. The detection of DnaK in culture supernatant. DnaK in cell lysate and bacterial culture supernatant of THB were analyzed by western blotting. The proteins were subjected to SDS-PAGE and were either stained with Coomassie Brilliant Blue (CBB) or transferred to an Immobilon-P membrane for western blotting using the anti-DnaK antiserum.

a) HUVECs

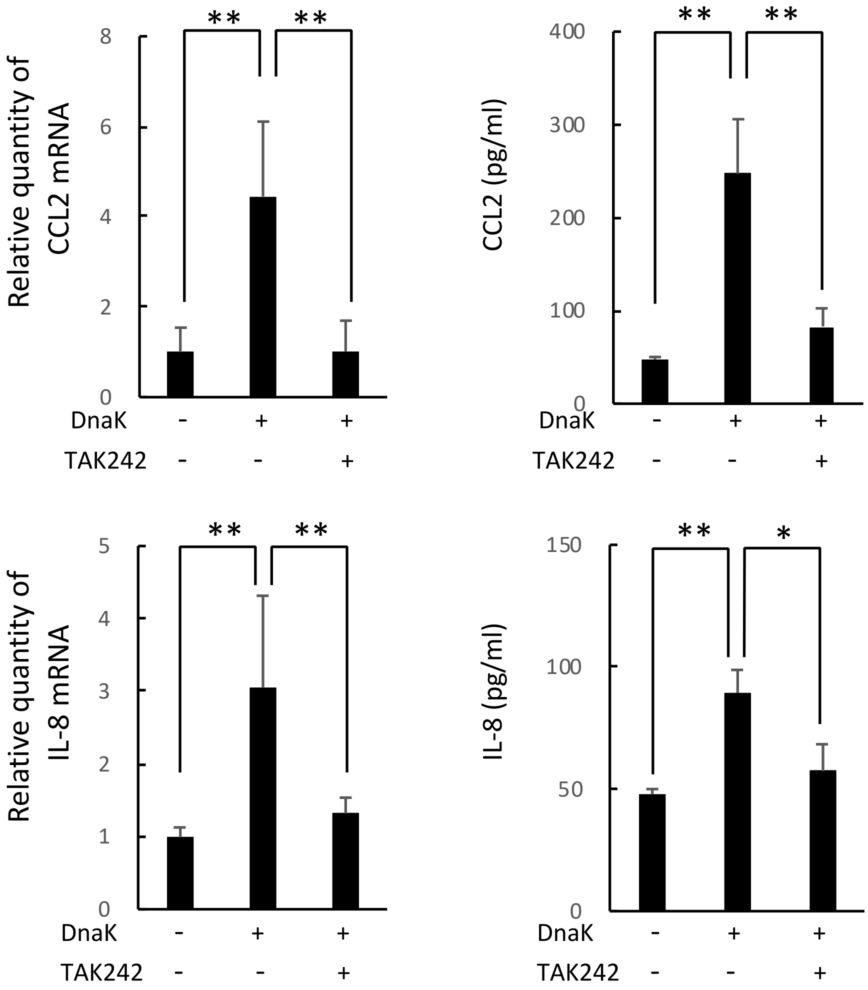

b) THP-1

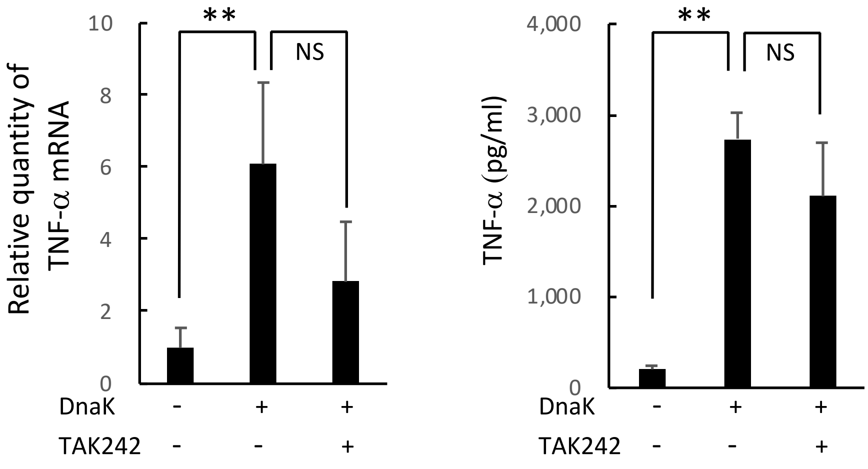

Figure S3. The effect of TAK242 on the activities of DnaK on HUVECs and THP-1 cells. HUVECs or THP-1 cells were pretreated with 1 μM of TAK242 (Cemi Science, Inc, QC, Canada), an inhibitor of TLR4 signaling, for 30 min at 37℃. Data are expressed as the mean ± SD from three independent experiments, each performed in duplicates. Statistically significant differences; $^{**}P < 0.01$, $^{*}P < 0.05$

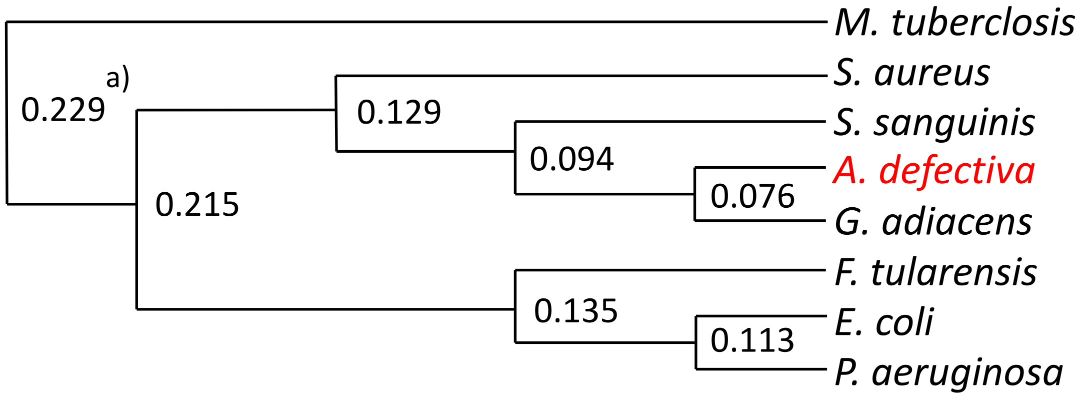

a) Omega score

Figure S4. Similarities among DnaK amino acid sequences from bacterial species as per the algorithms in the Clustal Omega multiple alignment tool.
